# Tiny-T: A small-scale demonstration tank for real-time wave energy control

**DOI:** 10.1016/j.ohx.2025.e00733

**Published:** 2025-12-20

**Authors:** Pedro Fornaro, Jacopo Ramello, Facundo Daniel Mosquera, Giuseppe Giorgi, John Vincent Ringwood

**Affiliations:** a Centre for Ocean Energy Research, Department of Electronic Engineering, National University of Ireland Maynooth, Ireland; b Marine Offshore Renewable Energy Lab (MOREnergy Lab), Department of Mechanical amd Aerospace Engineering, Politecnico di Torino, Italy; c Instituto de Investigaciones en Electrónica, Control y Procesamiento de Señales - LEICI (UNLP-CONICET), Facultad de Ingeniería, Universidad Nacional de La Plata, Argentina

**Keywords:** Wave energy, Real-time control, Demonstrator, Wave tank, Cost-effective, Low-cost alternative

## Abstract

This article presents a detailed description, and step-by-step process, required to build a small-scale wave energy tank demonstrator (Tiny-T). The system consists of an operative wave energy converter (WEC) with active control, representing the first demonstration of electronic WEC control at 1/100 scale. A key feature of Tiny-T is that the full demonstration system costs below €600. This document provides complete details on the materials, construction, and testing of the system, as well as aspects related to the appeal of the demonstration. As an experimental platform, Tiny-T represents a valuable resource for introductory research inquiries, catering to both high school and university-level studies. Overall, Tiny-T accessibility opens doors for a broader audience to engage with the promising potential of wave energy and real-time control technologies.

## Graphical abstract

**Figure:**
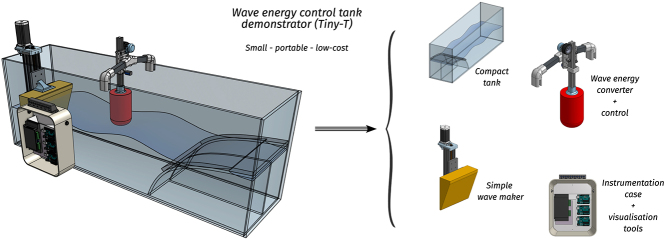

## Specifications table

**Table:** 

Hardware name	*Small-scale wave energy tank demonstrator (Tiny-T)*
Subject area	*Engineering and renewable energies*
Hardware type	*Electronic and mechanical engineering*
Closest commercial analog	*No commercial analog is available.*
Open source licence	CC BY 4.0
Cost of hardware	*€550*
Source file repository	Design files (DOI: 10.17632/hdp8njjrdn.2)

## 1 Hardware in context

Wave energy converters (WECs) represent an alternative renewable energy source that harness the kinetic or potential energy from ocean waves and convert it into usable electrical energy [1]. Although wave energy has not received the same levels of penetration as other renewables, in a context with worldwide societal interest in decarbonisation, wave energy technologies can play a fundamental role in contributing to, and diversifying, the energy mix and reducing the dependency on carbon-based energy sources. This is primarily due to two characteristic features of wave energy technologies. First, the possibility to complement other renewables: The high power density and reduced variability of waves can help to enhance grid stability, resilience, and grid penetration, hence also reducing the need for large energy storage systems [2]. Also, wave energy technologies represent an untapped source of renewable energy: Although the exact figure for extractable wave power remains debated [3], [4], it is estimated that wave energy possesses a global annual potential estimated between 1 and 10 TWh [3].

Despite their promising renewable energy technological potential, WECs face a variety of techno-economic challenges that hinder their widespread commercialisation [1], [5]. Primarily, economic limitations arise from high investment levels required to evaluate WEC prototypes, with a diverse plethora of diverse prototypes all vying for market leadership. Consequently, the apparent risk associated with investing in emerging wave energy companies results in an economic bottleneck, a phenomenon known as *the valley of death* [6]. On the technical side, the main problems are associated with developing robust and reliable technologies to withstand marine environments.

As a beacon of hope, control system technologies present an opportunity to solve both technological and economic limitations of WECs. First, by resorting to active control, it is possible to increase energy conversion by a factor of 2–3, with only a marginal increase in cost [7], [8]. Additionally, by keeping the system within physical constraints, control systems guarantee safe operation while maximising the WEC useful life. However, evaluating the effectiveness of such controllers on full scale devices, and sea conditions, remains a challenge. Initially, control performance may be assessed on smaller-scale prototypes, typically designed at earlier stages of development. Interestingly, although small/mid-scale testing and evaluation are well-established engineering practices, for WEC technologies, simulation tools (used at early-development stages) lack physical interaction, a critical component for understanding real-world dynamics (and are typically constructed using proprietary software), and second, testing facilities for WEC technologies (used at mid-development stages) are prohibitively expensive and primarily accessible only to large research institutions or corporations, representing an impediment for small-scale research projects or educational/demonstration purposes.

**Fig. 1:**
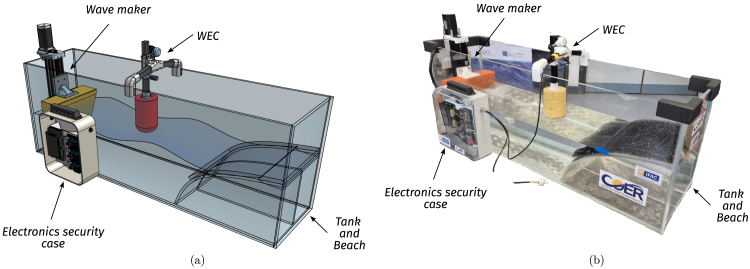
Tiny-T (a) Preliminary CAD design with system in operation. (b) First Tiny-T prototype.

In this context, this paper introduces a small-scale, cost-effective wave tank demonstrator (Tiny-T, illustrated in Fig. 1) designed to highlight the importance of wave energy as a promising renewable energy source and, fundamentally, to highlight the impact of control technology on power absorption. Furthermore, Tiny-T represents not only a dissemination and demonstration platform, but also an accessible research tool to conduct introductory and limited physical experimentation. In essence, Tiny-T is an impactful and cost-effective platform, developed with an open-source philosophy and offering a hands-on experience for exploring WEC control and wave energy fundamentals. By providing an accessible lower-fidelity alternative to large-scale wave tanks and purely computational tools, the system empowers researchers, educators, and students to investigate WEC behaviour under controlled wave conditions.

Tiny-T is designed with portability as a prime consideration, maximising the use of the tank in demonstration, education, and public engagement. However, the need for a low-cost and portable system results in inevitable compromises in terms of ease of control implementation and hydrodynamic fidelity. First, from a hydrodynamic perspective, in small tanks, two aspects limit the quality of the generated waves: The length of the tank, and the precision of the wavemaker [9]. Second, difficulties associated with implementing real-time control on a very small scale must be overcome. The actuation system must be fast enough to control the (faster) reduced-scale system dynamics, and non-linear mechanical (typically friction) and/or hydrodynamic viscous effects, which may be neglected for large-scale devices, are accentuated in small-scale devices and counteract the effects of control. However, a *minimal* tank size exists, in which realistic waves (with minimal presence of evanescent (transient) modes produced near the wavemaker) are realised, and real-time control can be effectively implemented. Specifically, Tiny-T is designed as a compact tank, balancing wave fidelity, control precision, and portability, while simultaneously providing a cost-effective and reproducible platform to allow wide dissemination of wave energy principles and, essentially, physical demonstration of the capability of real-time control in doubling energy capture.

In the remainder of the paper, the instructions required to replicate Tiny-T are presented and thoroughly detailed. First, Section 2 presents a description of the hardware and its composing subsystems. Section 3 then provides links to repository files with design specifications. Section 4 presents the bill of required materials, and Section 5 is a detailed set of instructions to construct the tank. Finally, Sections 6, 7 present the operation instructions and experimental validation results.

## 2 Hardware description

Tiny-T is a small-scale wave energy tank demonstrator (see Fig. 1). In essence, it comprises an easy-to-carry wave tank and a controlled point absorber-like WEC, complemented with sensors and instrumentation. The main features and specifications of Tiny-T are presented in Table 1.

In this section, each Tiny-T subsystem is thoroughly described. Following an open-source philosophy, modifications to the proposed design are welcomed and encouraged. However, it is important to note that a fundamental feature of Tiny-T is its affordability, which is achieved by designing and building it with off-the-shelf and easy-to-mount components, complemented with bespoke 3D pieces for subcomponent interconnection. In the remainder of the paper, 3D pieces are identified using the nomenclature “3D - Subcomponent acronym - Piece Number”, e.g., 3DWM01 is a 3D piece for the wave maker (WM), and identified as piece # 1.

**Fig. 2:**
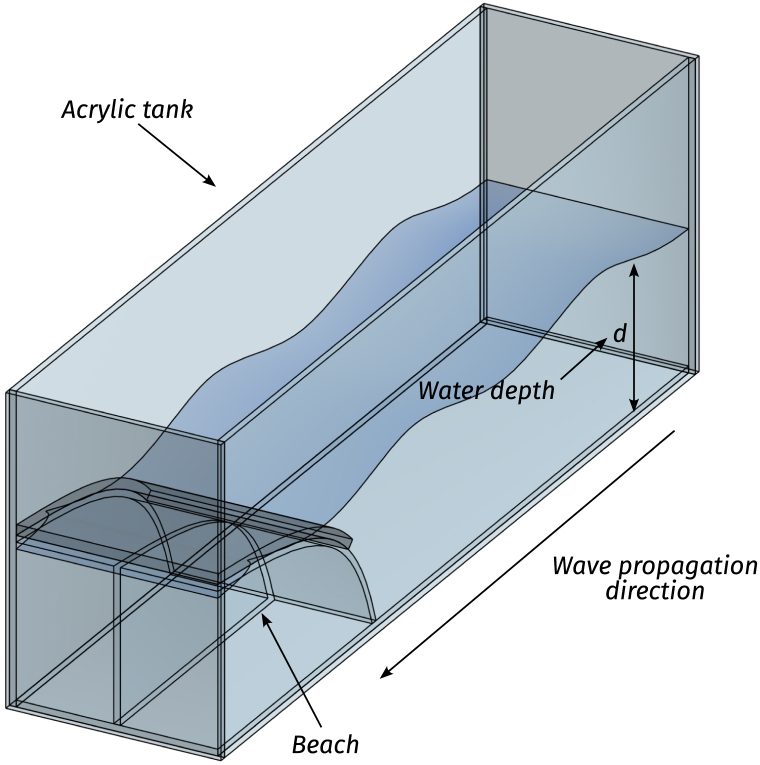
Illustrative model of the designed tank and beach.

**Table 1:** Tiny-T specifications.

Tiny-T main features		
Tank dimensions	• (L,W,H)	1300×300×450 [mm].
	• Nominal water volume	0,06 [m${}^{3}$]
	• Dry weight	25 [kg]
	• Nominal full weight	85 [kg]

Generated waves	• Type of waves	Sinusoidal
	• Period range	[0.6–1.6] [s]
	• Amplitude range	[0–22] [mm (peak-peak)]

Floating body	• Type	Point absorber
	• Control	Half-period latching [8], [10]
	• Resonance period	≈ 0.7 [s]

### 2.1 Tank and beach

At the heart of the system is the wave tank, a transparent acrylic structure (see Fig. 2) designed to balance ease of transportation with wave generation fidelity. The tank size ensures portability, while maintaining sufficient dimensions to generate and observe stable and regular wave patterns. Specifically, on the one hand, the tank is designed to fit in vehicles such as cars or standard-sized elevators, ensuring its portability. On the other hand, the tank dimensions (presented in Table 1) are sufficient to obtain monochromatic waves with relatively low harmonic distortion, allowing for introductory experimental research and laboratory demonstration.^1^

**Fig. 3:**
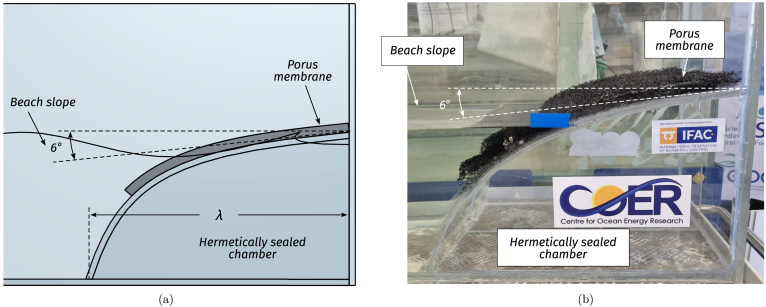
Beach figures. (a) CAD conceptual design. (b) Picture of the constructed tank beach.

Inside the tank, waves travel unidirectionally from the wavemaker towards a *beach*, included to effectively mitigate the occurrence of reflected waves [11]. The beach is a 53 [cm] long acrylic sheet, bent to obtain a slope of 6°at the tank boundary and a base length of one maximum wavelength (λ≈43cm), as illustrated in Fig. 3. Additionally, a highly porous mesh overlay is included over the beach surface to further prevent the propagation of high-frequency waves, enhancing the effectiveness of the wave-damping system. Below the mean water level, the beach angle is continuously increased, forming an arc shape that imitates the decreasing (with depth) circular motion amplitude of water particles. The goals behind the selected design are twofold: First, minimising the space required to build the beach, and second, since the beach interior constitutes an isolated chamber, it reduces the tank weight when filled with water, facilitating its transportation.

As for the employed materials, to curtail weight while retaining structural integrity, the tank structure, including walls and beach, is built using acrylic sheets and hermetically sealed with silicone glue. Importantly, acrylic provides the tank with durability and transparency. Although transparency is not strictly required for tank operation, it is highly recommended to visualise wave propagation, WEC motion, and the wave maker operation, which is essential to use Tiny-T as an effective demonstrator.

**Fig. 4:**
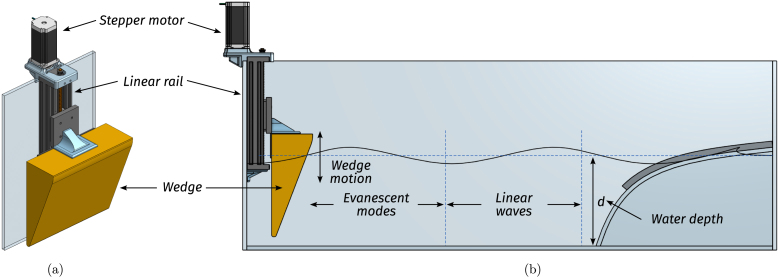
Wave maker illustrative figures (a) CAD design for the wave maker. (b) Illustrative diagram of the wedge-like wave maker in operation.

### 2.2 Wave maker

**Fig. 5:**
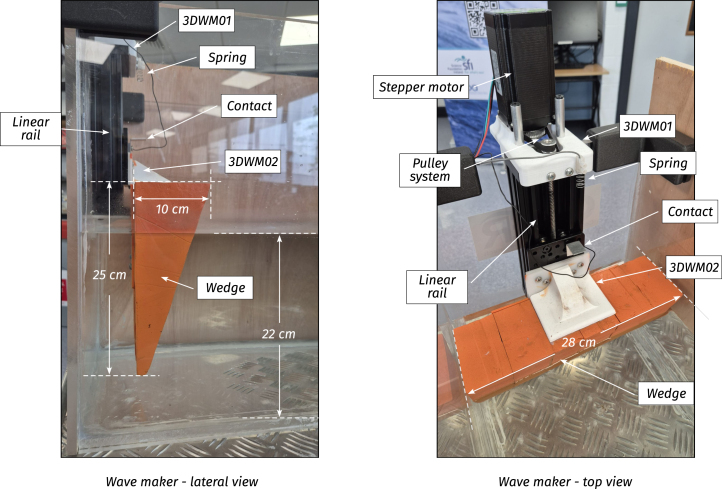
Designed wave maker. Each component used to design the wave maker is illustrated and presented following the code used in Table 2.

The wave maker (WM) produces waves in the tank. It comprises a triangular wedge and a controlled stepper motor, programmed to exhibit sinusoidal motion (see illustrative Fig. 4). The wedge material is high-density expanded polystyrene (HD-EPS), which minimises the load on the stepper motor, and the wedge dimensions are L=10 cm, H=25 cm, and a width W=28 cm, almost matching the full width of the tank, leaving slight clearance to prevent contact with the side walls and reduce additional hydrodynamic drag (see Fig. 5). On the other hand, the wedge motion is controlled using off-the-shelf 3D printer components: A z-axis linear rail and a stepper motor. For details regarding the employed components, the reader is referred to Table 2.

The adopted WM design [12], [13], compared to commercial solutions, sacrifices wave fidelity while prioritising essential features for demonstration purposes [14], as illustrated in the following. First, the WM is easy to operate, making it ideal for introductory research and demonstrations. Second, the required electronics are placed on top of the tank, away from corrosion and degradation caused by water immersion. Third, required to improve portability, the wedge-based WM is compact and occupies the minimum possible space. Alternatives, including flap- and piston-type wavemakers, occupy significantly more space and have higher maintenance requirements (e.g. dry back sealing, etc.) [15]. Furthermore, it is essential to highlight that, despite the anticipated sacrifice in terms of wave fidelity, the generated waves possess relatively low harmonic distortion (see Section 7), and a dynamic range that provides the user with flexibility to reproduce ad-hoc wave scenarios. Specifically, Tiny-T produces sinusoidal waves with an operational range defined by the stepper motor/linear rail, and the desired quality of the generated waves. First, the amplitude range is defined by the linear rail stroke, which is between [0,122.85]mm, enabling the generation of waves with amplitudes ranging between [0,2.2]cm. On the other hand, the period of the generated waves ($T_{w}$) depends on both the tank dimensions and stepper motor speed:

• The maximum allowed period (1.6s) creates wavelengths λ=L/3. As illustrated in Fig. 4, larger periods would create waves with non-vanishing evanescent modes at the WEC position.
• The minimum period (0.6s) is given by stepper motor limitations. A faster motor could potentially create waves with a higher frequency.

Importantly, the generation of irregular waves is not recommended, due to non-vanishing evanescent modes appearing near the floating structure position.

**Table 2:** Wave maker components specifications.

Wave maker		
Piece	Specifications	

Stepper motor	• Model	23HS45-4204S

Linear rail	• Model	Z axis 3D printer 4080U
	• Length	200 [mm]
	• Peace	8 [mm]
	• Stroke	122.85 [mm]

Pulley system	• Aluminium pulley	80 Teeth
	• Aluminium pulley	20 Teeth
	• Hole	8 [mm]
	• Belt	250-2GT

Wedge	• Material	HD-EPS
	• Dimensions (W, H, L)	28×10×25 [cm]

Auxiliary components	• 3D pieces	3DWM01–3DWM03

### 2.3 Point absorber WEC

**Fig. 6:**
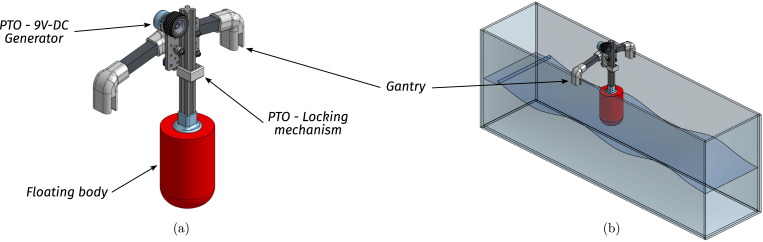
Illustrative image of the WEC mounted on the tank.

The point absorber WEC is a 1/100 scale and a simplified model of floating devices used in real-world wave energy systems (e.g. [16]). The WEC system is composed of three subsystems: A floating body (FB), a gantry (G), and a power take-off (PTO) mechanism (see illustrative Fig. 6). The WEC principle of operation is simple: The floating body oscillates with the reciprocating motion of waves, and, using active control and the PTO, the mechanical energy is transformed into electrical energy. The gantry, on the other hand, possesses the fundamental task of holding the FB in place and restricting its displacement to a single (heave/vertical) degree of freedom. In the following, each WEC subsystem is described.

**Fig. 7:**
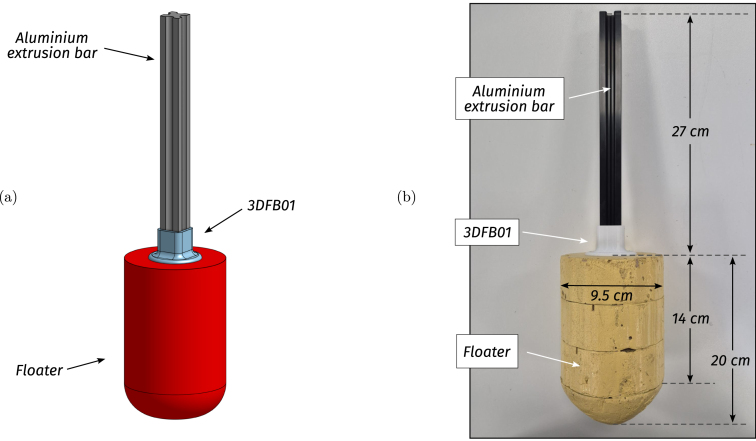
Floating body dimensions. (a) Preliminary CAD model. (b) Built prototype.

**Fig. 8:**
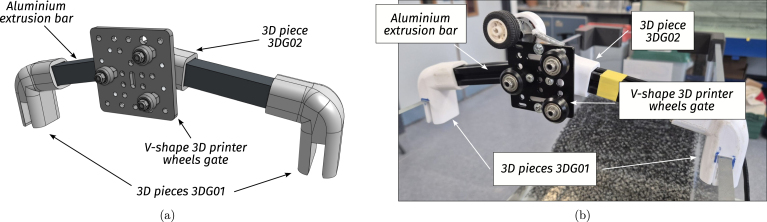
Gantry. (a) CAD illustration of the gantry. (b) Final built prototype.

**Fig. 9:**
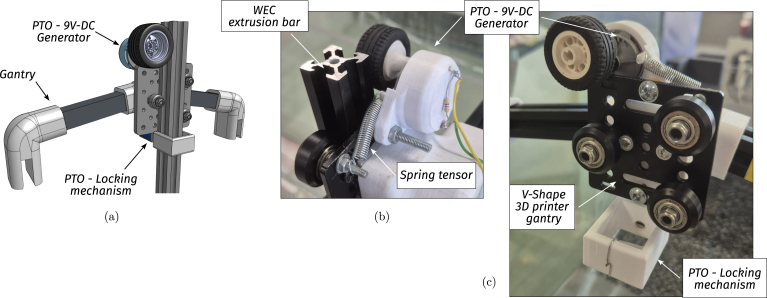
PTO detail. (a) Front view - CAD illustration of the PTO elements. (b) Back view - Detail of the contact between the extrusion bar and the LEGO wheel. (c) Front view - 9V-DC generator and locking mechanism mounted on the gantry.

**Fig. 10:**
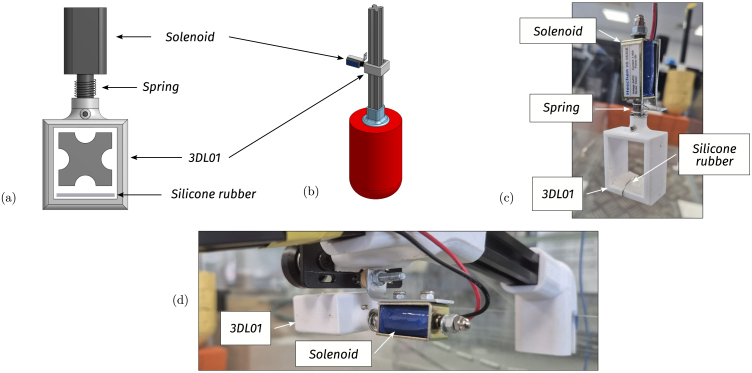
Illustrative image of the locking mechanism.

• **WEC floating body:** The floating body comprises a floater connected to the PTO via an aluminium extrusion bar. The floater is built using HD-EPS, which has high buoyancy properties, and the aluminium bar adds weight to the floater and serves as a link between the FB and the PTO (see Fig. 7, Fig. 9.a). In essence, the FB is a resonator, and its dimensions determine the resonance frequency (alternatively, period). The FB size is designed considering that the employed control strategy (see Section 2.4) requires the FB resonance period $T_{d}=2\pi /\omega_{d}$, to be shorter than the wave period, $T_{w}$ (the employed control philosophy, *latching*, can only slow the device down). The adopted FB dimensions are concisely summarised in Table 3 and illustrated in Fig. 7.
• **Gantry:** The gantry body is composed of two aluminium extrusion bars and 3D pieces (illustrated in Fig. 8, detailed in Table 4). The lateral 3D pieces of the gantry facilitate easy mounting/dismounting, and the central piece of the gantry serves as a support for the remainder of the elements in the WEC system, namely, the floating body and the PTO. From a WEC operation perspective, the gantry has a dual purpose. On the one hand, the gantry limits the FB operational space to prevent drifting and, on the other hand, the gantry confines the FB motion to be predominant in the heave (z-axis/vertical) direction. Importantly, the gantry should have minimal friction, to facilitate displacement of the FB along the z axis. To achieve this, the central piece is designed using 3D printer components. Specifically, a V-shape 3D printer wheel gate with bearings is used. The wheels in the central piece of the gantry engage with the side guides of the aluminium extrusion bar of the FB, reducing friction levels while limiting displacement to the z axis (see Fig. 8 and, alternatively, Fig. 9).
• **Power take-off (PTO):** The PTO mechanism consists of two separate systems. The first component is a 9V-DC generator, generating electrical power from vertical movement of the FB (see Fig. 9). The second is a locking mechanism, or brake, designed to hold the FB in place during specified time intervals (see Fig. 10). Both PTO components are designed and selected considering affordability and practicality, to reduce mounting complexity to a minimum, and enhance overall system robustness and durability. The generator consists of a LEGO wheel and a 9V-DC motor and is encased in a 3D printed shell, mounted on top of the gantry using spring tension to ensure good contact with the WEC-buoy aluminium extrusion bar (for details, see Figs. 9.b and 9.c). Additionally, a LEGO gear is included to increase generator speed (and, therefore, efficiency) and provide sufficient output voltage. On the other hand, the locking mechanism consists of a solenoid and a brake, built with a 3D piece and silicone rubber (see Fig. 10), and mounted under the gantry with a simple screw-nut and a metal plate. Importantly, after positioning the solenoid, fine-tuning is required to ensure, considering the braking distance, that the locking mechanism effectively holds the FB in place. Technical specifications for the employed PTO components are presented in Table 5.

**Table 3:** WEC floating body specifications.

WEC Floating body		
Piece	Specifications	

Floater	• Diameter	95 [mm]
	• Height (cylindrical part)	140 [mm]
	• Total height	200 [mm]
	• Material	HD-EPS

Bar	• Aluminium extrusion bar	2020 profile
	• Length	27 [cm]
	• Auxiliary 3D piece	3DFB01

**Table 4:** Gantry component specifications.

WEC gantry		
Piece	Specifications	

Bar	• Aluminium extrusion bar	2020 profile
	• Length	22 [cm]

Connectors	• 3D press-fit joints	3DG01 - 3DG02
	• Gantry centre piece	V-shape 3D printer wheels gate (with bearings)

**Table 5:** PTO components specifications.

PTO components		
Piece	Specifications	

Break	• Solenoid model	HS-0530B DC6V (Push-pull)
	• Solenoid stroke	10 [mm]
	• Solenoid force	5 [N]
	• 3D Piece	3DL01

Generator	• 3D pieces	3DM01–3DM02
	• LEGO motor model	71427c01 (9V-DC Mini-Motor)
	• LEGO wheel model	4 184 286 (43.2 × 22 ZR)

### 2.4 Micro controllers and ancillary electronics

In addition to the components presented in Sections 2.1, 2.2, 2.3, to enable real-time monitoring and control, Tiny-T integrates sensors and electronics, separately presented below. Following an open-access philosophy and targeting high system replicability, off-the-shelf components (such as Arduino UNO microcontrollers) are used. However, if desired and accordingly with their availability, the user may employ different microcontrollers and components.

**Fig. 11:**
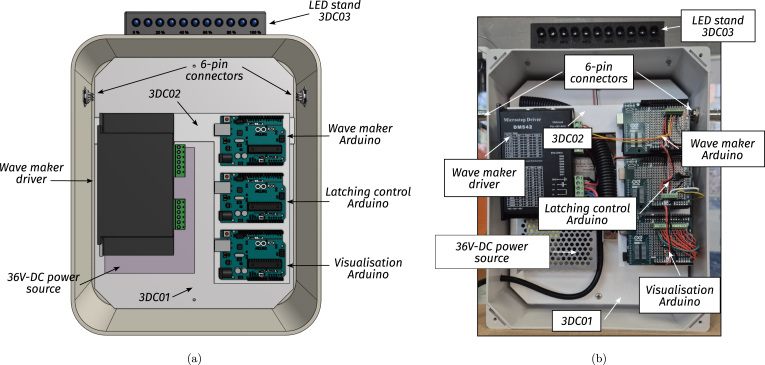
Electronics case. (a) Illustrative design. (b) Built electronics case.

**Fig. 12:**
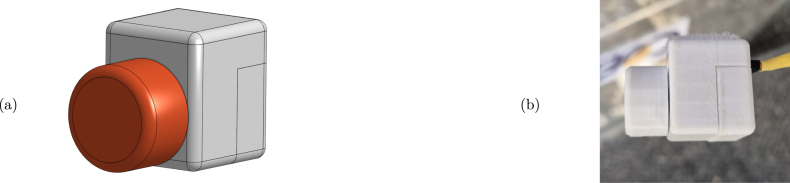
User-accessible control knob. (a) Illustrative design. (b) Built user knob.

• **Wave maker instrumentation:** To control the wave maker, an Arduino UNO is used to command a stepper motor drive. Additionally, an end-stop switch, consisting of a metal spring and plate, is included in the wave maker and used as an input signal for the wavemaker Arduino. To easily mount/dismount the Arduino, the connections are made using an Arduino Proto Shield (see Fig. 11.b). The code required to program the wavemaker Arduino and the connection schematics are available for download through the link provided in Section 3, together with the Proto Shield schematic.
• **PTO instrumentation:** The PTO components, namely the generator and locking mechanism, are connected to two Arduino UNO devices using two external Proto Shields and external instrumentation. The first Arduino board is in charge of controlling the locking mechanism, using a transistor-based trigger mechanism. The second Arduino (visualisation Arduino) measures output voltage data from the 9V-DC LEGO wheel and computes its RMS value to provide and display, using an LED array, a *relative* measure of the WEC absorbed power. For details regarding the normalisation required to use voltage measurements as surrogates of power output please refer Section 7.3.
  The files required to program both Arduinos are available in the links provided in Section 3, together with the Proto Shield schematics.
• **Power sources:** Each Tiny-T component requires a stable DC power source. Specifically, critical components are the stepper motor and driver. To comply with the motor and electronics requirements, two separate DC voltage sources (detailed in sections 3 and 4) are used and mounted inside a security case.
• **Electronics casing:** Each electronic component must be isolated from possible contact with water. Hence, as presented in Fig. 11, the electronic instrumentation, including the required power sources, the Arduinos, and additional PCBs, is encased and neatly presented in a transparent box that permits visualising of the connections, further enhancing user curiosity. The connections between the wavemaker, WEC, and PTO with the case are made using two 6-pin connectors.
  By using the 6-pin connectors, it is possible to mount/dismount the electronics from the tank while reducing security hazards. Importantly, following the design proposed for Tiny-T, every electronic component remains above or outside the tank and securely isolated.
• **Real-time control and visualisation tools:** Since one of the main functions of Tiny-T is to highlight the effect of control on energy absorption, real-time control represents an essential feature. Specifically, a latching strategy [8] is selected to show the efficacy of control strategies to increase power output. Essentially, latching is a classic WEC control strategy that consists of holding the device still to reduce (increase) the natural WEC FB resonance frequency (period) to match the frequency (period) of incoming waves. The effectiveness of latching depends on several parameters, one of which is the *latching time*, namely the amount of time during which the device is locked. Thus, to tune the latching control, a linear potentiometer is placed outside the tank and encased in the 3D piece 3DUK01 (see Fig. 12). The potentiometer is connected to the latching control Arduino, which, in turn, modifies the *latching time*, providing a user-interactive platform to assess control performance.
  Additionally, to visualise the effect of varying the latching time, an LED array is placed on top of the tank electronics case. The LED array is used to indicate a *relative* measure of the average power absorption increase ranging from 0% to 100%, where 0% represents no power absorption, and 100% represents the case where the optimal latching time is selected, for which maximum power absorption is attained. The estimate for the relative average power output is obtained by computing the 9V-DC motor RMS output voltage and normalising it with an estimate of the (constant) RMS output voltage obtained when the optimal latching time is selected. That is, the percentage of power absorption displayed in the LED array, $P_{\text{\%}}$, is
  (1) $$P_{\text{\%}}=\frac{v_{rms}}{v_{rms|max}},$$
  where $v_{rms|max}$ may be obtained empirically as detailed in Section 7.3. Hence, values between 0% and 100% are obtained by using a suboptimal latching time or no control (For further details regarding the energy monitoring process, please refer to Section 7.3).
• **Additional sensors:** To further complement Tiny-T, additional sensors could be included. For instance, to acquire wave elevation data, capacitive or resistive wave probes [17] may be used. It is important to note that, with the *minimal* dimensions of Tiny-T, wave probes may only be used to characterise the undisturbed wave maker operation, and placed (removing the FB) at the gantry position. Additionally, recall that wave probes, together with the required instrumentation, would significantly increase the cost of Tiny-T.
  Also, depending on the desired data, position/velocity sensors may also be included (see recommended sensors in Table 6). Optical sensors, specifically, can be placed either in top or below the gantry by using the 3D piece 3DG02 and wired to an external microcontroller. Ultimately, the type and variety of sensors included depend on the desired use for Tiny-T. For demonstration purposes, for instance, solely employing the output voltage from the PTO is sufficient to illustrate the effect and impact of control in terms of energy absorption.

**Table 6:** Ancillary electronics specifications.

Ancillary electronics		
Piece	Specifications	

General	• Micro controller	Arduino UNO
	• Resistors	Various 1/4 W

WM instrumentation	• Motor controller model	DM542 (Dir-pul control)
	• Motor controller current peak	4.2 [A]
	• Contact	Metal plate and spring

PTO instrumentation	• Transistor	Darlington

Power sources	• Arduinos and WM controller	5 [V]
	• Stepper motor	36 [V]

Security case	• Waterproof junction box	245 × 305 × 125 [mm]
	• 3D piece	3DC01–3DC02
	• Connectors	GX16 (6-pin)

Control & Visualisation	• Potentiometer	10 [kΩ] linear (knob)
	• 3D piece	3DUK01 (knob)
	• LEDs	5 mm - Blue (visualisation)
	• 3D piece	3DC03 (visualisation)

Additional sensors	• Position sensor	VL6180X (time-of-flight sensor)
	• Wave elevation sensor	Churchill - Wave monitor with resistive wave probes.

### 2.5 Tiny-T uses and applications

By simplifying the complexity of large-scale systems, Tiny-T provides an interactive platform for exploring the fundamental concepts of wave energy conversion. Importantly, Tiny-T highlights the importance of control strategies, showing that, by a marginal increase in the system complexity, it is possible to synchronise the FB motion with incoming waves, maximising energy output. In the following are presented the main uses and applications of Tiny-T, while also highlighting the operational limitations of the built prototype. First, it is possible to summarise the main purposes for which Tiny-T may be used as follows:

• **Dissemination purposes**: Due to the high visual impact and efficacy of the simple control strategy, it can capture the attention of people of different ages and backgrounds, representing a powerful dissemination tool. As illustrative examples, Tiny-T has already been used in a conference (as part of a tutorial workshop and also exposed in the conference foyer), in public engagement events, and for high school visits (See media available through the link provided in Section 3).
• **Introductory research purposes**: Here, caution must be exercised. Due to the dimensions of Tiny-T, viscous effects, evanescent modes, and unmodelled nonlinearities in the 9V-DC generator and in the gantry bearings must not be neglected. However, as presented in Section 7, Tiny-T dimensions are sufficient to prove the proficiency of control. Subsequently, since the rig is highly customisable, it offers the possibility to conduct hands-on *limited* and introductory experimental tests in a realistic environment and evaluate the efficacy of different control strategies, and WEC bodies and shapes.
• **Educational purposes**: The platform may also be used, not only to explore wave energy principles, but also for teaching purposes for different areas ranging from energy conversion principles, coding, control, design, and various other engineering disciplines.

For any of the above-mentioned categories, the balance between accessibility and functionality ensures that Tiny-T can play a pivotal role in advancing knowledge and fostering interest in renewable energy technologies.

## 3 Design files summary

In the following, bespoke design files and schematics required to build Tiny-T are presented in Table 7. In Table 7, the required files are subdivided into six categories, described below. Importantly, since the files are not protected, the user may download and edit the provided material.

• In the folder 3D_designs, CAD files with the 3D designs for Tiny-T are available.
• The folder code contains the .ino files used to program each Arduino UNO board.
• The folders datasets, datasheets, and supplementary_material contain supplementary files, including design schematics, datasheets, raw datasets collected during Tiny-T validation, and photos included to assist the user for the replication of Tiny-T.
• As a complement for educators, the matlab_model_latching folder contains an illustrative example for latching control applied to a single-degree-of-freedom WEC model.

**Table 7:** Design files.

Design filename	File type	Folder	Access
3DG01.stl – 3DG02.stl	STL	3D_designs	Design files
3DFB01.stl			
3DL01.stl			
3DM01.stl – 3DM02.stl			
3DC01.stl – 3DC03.stl			
3DWM01.stl – 3DWM03.stl			
3DUK01.stl – 3DUK03.stl			

latcing_control.ino	Arduino code	arduino_code	
wave_generator.ino			
wave_probe.ino			

README	Text file	datasets	
latching.txt			
no latching.txt			
still water_new.txt			
wave_amp_t15s.txt			

data_processing.m	MATLAB file		
data_processing_dundalk.m			
dundalk_data.mat			

57A3 stepper motor.pdf	PDF	datasheets	
Datasheet 1n4001.pdf			
Datasheet JS-0530B solenoid.pdf			
Datasheet irf520.pdf			
dm542 user_manual.pdf		

Building schematics.pdf	PDF	supplementary_material

latch.m	MATLAB file	matlab_model_latching
latch2_2017.slx		

## 4 Bill of materials

In Table 8, the complete list of required materials to build Tiny-T is presented.

**Table 8:** Bill of materials.

Designator	Component	Quantity	Cost p/u [€]	Total cost [€]	Source of materials	Material
General	PLA wire	1	17.91	17.91	Online stores	Plastic
	Heat shrink	As needed	5	5	Online stores	Plastic
	Cable	As needed	5	5	Online stores	Metal
	Metal plate	1	0.05	0.05	Hardware stores	Metal
	Screw-nuts	As needed	5	5	Online stores	Metal
	Spring	3	0.4	1.2	Online stores	Metal
	Foam	1	5	5	Hardware stores	High density stereo-foam
	2020 extrusion bar	1	25.99	25.99	Online stores	Metal

Wave maker	Pulley 80-20 T	1	16.00	16.00	Online stores	Metal
	Neema 23 step motor	1	44.84	44.84	Online stores	Metal
	CNC z axis linear actuator	1	92.18	92.18	Online stores	Metal
	DM542 step motor controller	1	24.28	24.28	Online stores	Semi-conductor

Tank	1 cm Acrylic sheets	5	150 ca	150 ca	Hardware stores	Acrylic
	0.5 cm Acrylic sheets	1	15 ca	15 ca	Hardware stores	Acrylic
	1 cm plastic mash sheets	1	5 ca	5 ca	Hardware stores	Plastic

PTO & Gantry	V-shape 3D printer gantry	1	13.76	13.76	Online stores	Metal
	5 V-5 N solenoid	1	8.49	8.49	Online stores	Metal
	LEGO wheel and 9 V-DC motor	1	6.5	6.5	Online stores	Metal

Security case	Arduino UNO	3	10.85	32.55	Online stores	Semi-conductor
	Arduino UNO protoshields	3	2	6	Online stores	Plastic
	Waterproof junction box	1	7	7	Online stores	Plastic
	6-pin connector	2	7	7	Online stores	Plastic
	Power supply 36 V	1	32.99	32.99	Online stores	Semi-conductor
	Power supply 5 V	1	17.99	17.99	Online stores	Semi-conductor
	6-Pin GX16 connector	2	1.3	2.6	Online stores	Plastic/metal
	5 mm LED	10	0.2	2	Online stores	Semi-conductor
	5 mm LED holder	10	0.1	1	Online stores	Plastic

## 5 Build instructions

In the following, design and building instructions for each Tiny-T subsystem are presented, along with illustrative diagrams.

### 5.1 3D components

1. Download the 3D design files from the link provided in Section 3.
2. Print each 3D piece. For Tiny-T, nominal specifications for PLA were used, with a 10% fill density to curtail expenses. However, the user is encouraged to evaluate the amount of material used and the precision of the 3D printer.

### 5.2 Tank & beach

**Fig. 13:**
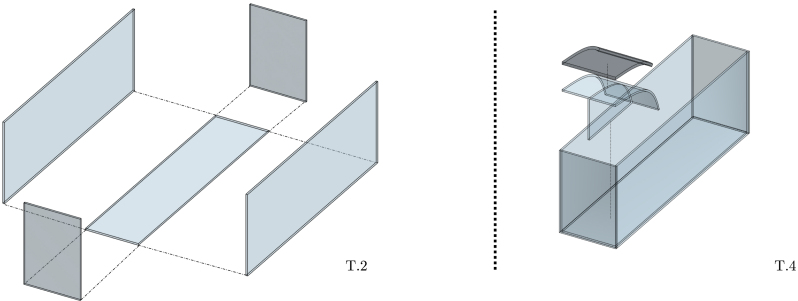
Assembly of the tank and the beach.

To build the tank and beach, follow the procedure described above. An illustrative diagram is presented in Fig. 13.

T.1. Carefully cut the acrylic sheets. A saw with fine-tooth blade may be used, cutting slowly to prevent melting edges.
T.2. Glue the tank laterals and base, one by one (See illustrative Fig. 13), by using silicone glue to prevent leaking. The original prototype is built using regular neutral silicone glue, since the water volume does not reach critical hydrostatic pressure limits that could cause tank leakage. However, water-thin solvents can also be used to enhance tank durability. On the other hand, acetoxy silicones, cyanoacrylate, epoxy resins, or polyurethane adhesives are not recommended.
T.3. Design the beach end, using a heat gun to bend the acrylic sheet.
T.4. Using screws and glue, attach the curved beach to the auxiliary acrylic support placed inside the beach chamber. See illustrative Fig. 13.b.
T.5. Carefully glue the beach to the interior part of the tank, adding neutral silicone on the junctions and edges to hermetically seal the beach interior chamber.
T.6. Finally, place and glue the porous material on top of the beach acrylic sheet, also using silicone glue or acrylic solvent cement.

### 5.3 Wave maker

To build the wave maker, follow the steps detailed below.

**Fig. 14:**
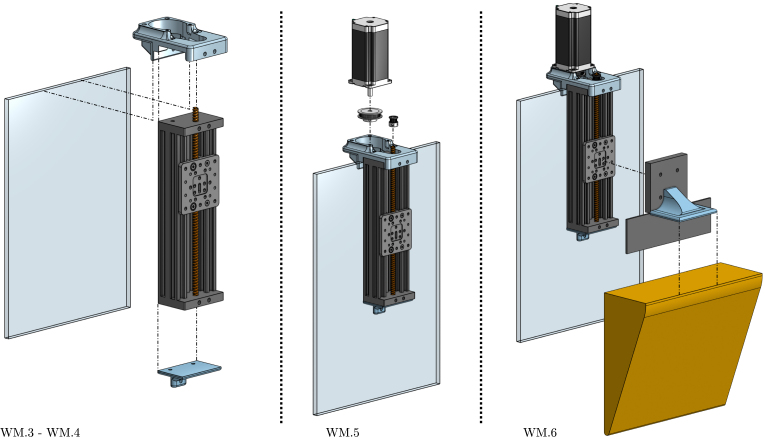
Assembly of the wavemaker.

WM.1. Cut HD-EPS to build the wave maker wedge using the dimensions specified in Section 2. Consider that, to glue the HD-EPS parts, foam-safe adhesives must be employed (wood glue, water-based contact cements or polyurethane adhesives).
WM.2. If desired, paint the wedge with non-corrosive paint.
WM.3. Place the 3D pieces 3DWM01-3DWM03 on the top and bottom of the rail (see Fig. 14).
WM.4. Carefully place and glue the wave maker linear rail inside the tank. For Tiny-T first prototype, neutral silicone was used together with water-thin solvent cement. Through holes with screw-nuts and bolts could also be used; however, special considerations must be made not to damage the acrylic sheets.
WM.5. Place the pulley components and fix the stepper motor to piece 3DWM01 using screw-nuts and bolts.
WM.6. Finally, as presented in Fig. 14, connect the wedge to the linear rail using the 3D piece 3DWM02 and screws, which facilitates easy mounting/dismounting of the wedge.

For further details, the specifications for the elements used to build the wave maker are presented in Table 2, and CAD files for the auxiliary 3D components can be found in the links provided in Section 3.

### 5.4 WEC, gantry and PTO

To build the WEC, gantry, and PTO, follow the procedures described below.

**Fig. 15:**
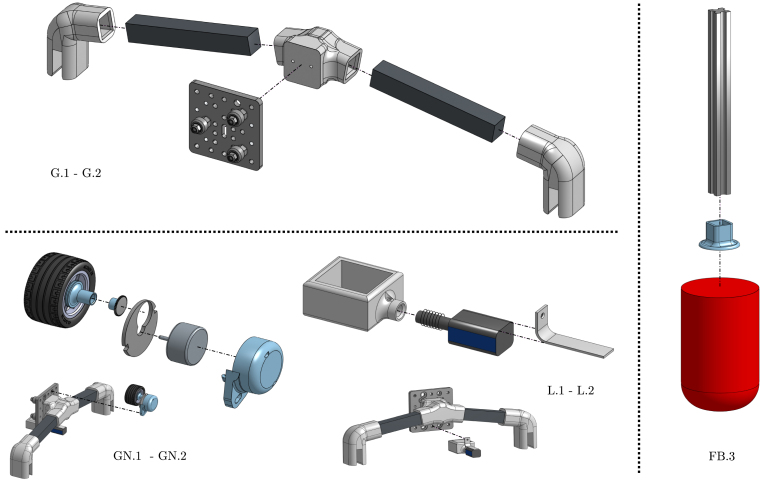
Illustrative diagram to build and mount the WEC FB, gantry, and PTO components.

• **WEC floating body** (see Fig. 15):
  FB.1. Cut and glue HD-EPS parts to design the floater with the dimensions presented in Section 2. Consider that, to glue the HD-EPS parts, foam-safe adhesives must be employed (wood glue, water-based contact cements or polyurethane adhesives).
  FB.2. If desired, paint the floater with non-corrosive paint.
  FB.3. Carefully position and glue the extrusion bar and the floater to the 3D piece 3DFB01. The floater must be glued using non-corrosive adhesives, while, to glue the extrusion bar to the 3D piece, cyanoacrylate (super-glue) may be used.

• **Gantry** (see Fig. 15):
  G.1. Glue the extrusion bars with the 3D pieces 3DG01 and 3DG02. Here, cyanoacrylate (super-glue) may be used.
  G.2. Place and fix the V-shape 3D printer gantry to the 3D piece 3DG02 using screw-nuts and bolts.
  G.3. Mount the gantry on the tank at the desired position (this is a flexible parameter for user experimentation).

• **PTO locking mechanism** (see Fig. 15):
  L.1. Screw the 3D piece 3DL01, used for the brake, to the solenoid. Note that, to release the braking mechanism, an external spring is placed between the solenoid and the brake. Fine tuning of the used spring is recommended to improve the latching operation.
  L.2. Using an external metal plate, fix the locking mechanism below the gantry. Importantly, after positioning the solenoid, fine-tuning is required to ensure, considering the braking distance, that the locking mechanism effectively holds the FB in place when latching control is used.

• **PTO generator** (see Fig. 15):
  GN.1. Place the 9V-DC LEGO generator inside the 3D piece 3DPT01.
  GN.2. Add the LEGO wheel and gear, and place the generator on top of the gantry using screw-nuts and bolts.
  GN.3. Add an external spring tensor to secure contact between the WEC extrusion bar and the generator. Note that, excessive contact between the LEGO wheel and the extrusion bar would result in high damping levels, hampering WEC displacement and, subsequently, power output.

### 5.5 Micro controllers and ancillary electronics

**Fig. 16:**
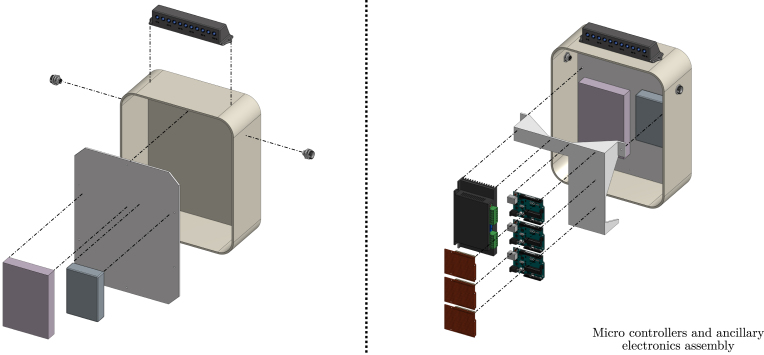
Illustration of the ancillary electronics position inside the security case.

The ancillary electronics are included in Tiny-T following the following procedures, illustrated in Fig. 16. Importantly, before mounting the components inside the security case, the user is encouraged to test each component operation individually.

**Fig. 17:**
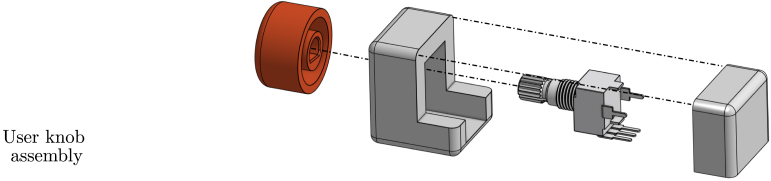
Illustrative diagram to build and mount the latch tuning knob.

• **Power supply and casing:**
  1. Place the auxiliary 3D printed piece 3DC01 inside the security case.
  2. Add two 6-pin connectors on each side of the security case.
  3. Using screws, mount the DC power sources to the 3DC01 piece and connect their outputs accordingly.
  4. Place the auxiliary 3D piece 3DC02 and mount the stepper motor controller. Wire accordingly, connecting the stepper motor controller output to the corresponding 6-pin output.
  5. Solder the visualisation LEDs and place them, using LED holders, inside the 3D piece 3DC03.
  6. Mount the 3D piece 3DC03 on top of the security case using through holes and screw-nuts.
• **Arduino UNO boards:**
  1. Using Arduino UNO Proto Shields, solder each component, following the schematics available through the link provided in Section 3.
  2. Mount the three Arduino UNOs on top of the 3DC02 piece.
  3. Mount the Proto Shields and connect each Arduino UNO accordingly.
• **Wiring:**
  1. Place the wavemaker cables inside cable sleeves and solder them to the corresponding 6-pin connector. If desired, use thermal shrinks to enhance security.
  2. Similarly, place the PTO cables, corresponding to the locking mechanism and generator, inside cable sleeves and solder them to the corresponding 6-pin connector.
  3. As presented in Fig. 17, assemble the user-accessible (latch timing) knob and solder the terminals to the corresponding 6-pin input.

## 6 Operation instructions

### 6.1 Step-by-step guide

To operate Tiny-T, follow the steps detailed below.

• Transportation and placement.
  1. To transport the tank, it is recommended to use a tank stand with wheels.
  2. It is recommended to transport the tank before it is filled.
  3. Consider the surroundings and, if possible, place the tank away from any external electronic devices.
• Tank setup.
  1. Before filling the tank with water, remove the gantry and WEC.
  2. Fill the tank by using a hose or bucket to the nominal water level d=22 cm. It is recommended to fill the tank using the beach end to prevent splashing.
  3. For higher visual impact, it is also possible to add non-contaminant water colourant (e.g. blue).
  4. Place the gantry in position at 70 cm from the wavemaker, and place the PTO inside the tank.
  5. Position and connect the electronic case to the wave maker and PTO components.
• Tank operation.
  1. Turn on the power source. This initialises the Arduinos; then Tiny-T will operate accordingly with the parameters defined in each Arduino UNO, until power is disconnected.
  2. Use the external knob to adjust the latching time (control parameter).
  3. After the experiments are finalised, turn off the power source (the tank operation stops immediately). If the Arduinos are connected to a PC, it is also necessary to disconnect them to turn off the tank.
  4. After using Tiny-T, the tank can be emptied using a (syphon) hose or bucket.
• Additional visualisation tools and supplementary content.
  1. It is possible to connect the Arduinos included in the electronics case to a PC to visualise the variables in the system. Additionally, position, velocity, and wave elevation may also be measured by using external sensors and displayed to provide additional visual and easy-to-interpret information.
  2. Different wave maker shapes may be used to evaluate the quality of the generated waves. To change the wave maker wedge, turn off Tiny-T and replace the desired wedge shape.
  3. While also increasing Tiny-T complexity, the interested user may evaluate the inclusion of different internet-of-things (IoT) features. Wireless connection to control/measure data could improve the user interface and further enhance user curiosity for public demonstrations.

### 6.2 Safety hazards

All electronic components must be kept dry inside the electronic case. In the event of malfunction (e.g., loose wiring or motor overextension beyond the limits of the linear guide), immediately disconnect power and inspect the system for faults. Additionally, conduct regular inspections on the tank to ensure there is no water leakage.

## 7 Validation and characterisation

In this section, the nominal Tiny-T operation is validated through real-time evaluation. The performance of Tiny-T is evaluated considering (a) the quality of the generated waves at the nominal frequency, (b) the effectiveness of real-time control in maximising power absorption, and (c) the suitability of the visualisation tools, required to use Tiny-T as a demonstration platform. In this regard, considering that the main use for Tiny-T is as a demonstrator for wave energy principles, tests are conducted at a single frequency, for which Tiny-T exhibits nominal performance. By conducting complementary research, the interested developer may assess Tiny-T operation outside its nominal operating range.

In addition to the results presented in this section, the interested reader may also refer to the supplementary material recorded and uploaded to the repository provided in Section 3, where it is possible to appreciate Tiny-T in operation.

### 7.1 Wave fidelity

To evaluate sinusoidal wave fidelity, a resistive wave probe was installed in the tank and used to measure wave elevation (see Table 6) with a sampling period of 7.8 ms. The measured variables are post-processed using Matlab to remove measurement noise. Due to the characteristics of the tank design, it is possible to assume that Tiny-T produces waves with high reproducibility. Then, the acquired data is evaluated in both time and frequency domains. Note that, to measure the generated waves, the WEC FB is removed and the resistive probe is installed in its place.

**Fig. 18:**
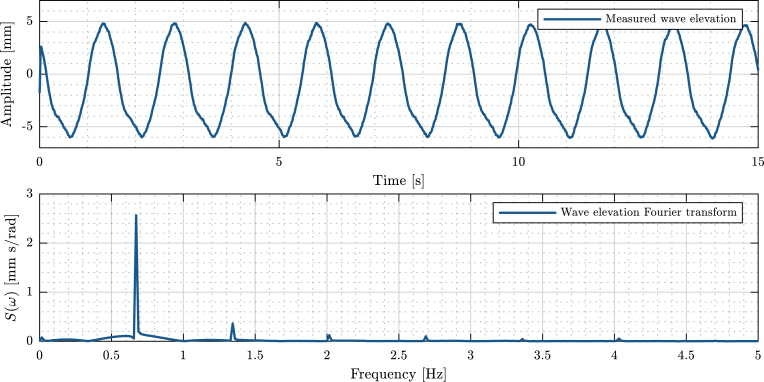
Wave elevation data. (a) Time evolution of the wave elevation. (b) Fourier transform of the wave elevation.

In this illustrative example test, the WM stepper motor is programmed with a 65 mm stroke and a fundamental period of 1.5 s, resulting in a wave amplitude that oscillates around 11 mm peak-peak (See Fig. 18.a). Importantly, as appreciated in Fig. 18.b, the wave elevation is sinusoidal with a small amount of harmonic distortion. The fundamental frequency peak is clearly evident at the desired frequency of .66 Hz (equivalently, $T_{w}\approx 1.5$ s). A small second and third harmonics can be observed.

In addition, to observe the wave patterns generated, the reader is referred to the videos available through the link provided in Section 3.

### 7.2 Position and velocity measurements

Tiny-T is, primarily, a demonstrator tank. Hence, evaluating the amplification of both position and velocity when control is applied, is essential. Motion amplification has two correlated effects. First, it creates visual impact, and second, it increases power output, which illustrates the effect of control from the perspective of the application as a source of renewable energy. Following Section 2.4, to measure position and velocity, an auxiliary VL6180X sensor (see Table 6) is mounted on the gantry, and position measurements are collected using an Arduino UNO. Also, in this illustrative example, the WM stepper motor is programmed with a 50 mm stroke and a fundamental period of 0.9 s.

**Fig. 19:**
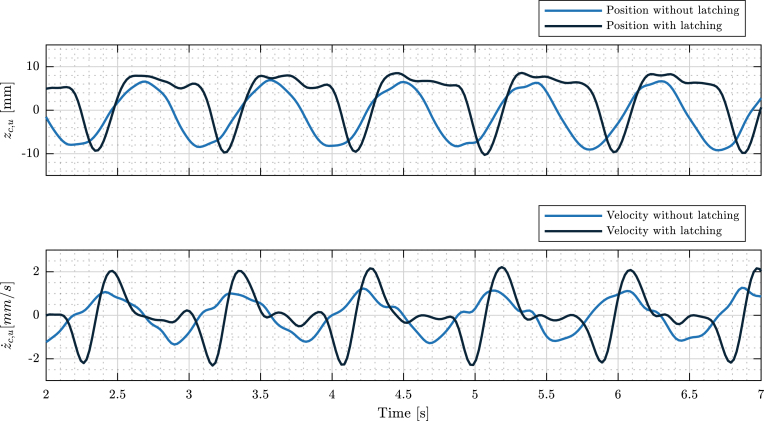

In Fig. 19, the controlled and uncontrolled FB position and velocity are presented. The effect of latching control in the WEC position is presented in Fig. 19.a, where it can be appreciated that the position peak is, approximately, 1.3 times the maximum displacement of the uncontrolled case (from 14 mm-pp to 18 mm-pp). Also, in Fig. 19.b, it is possible to appreciate that, although latching control holds the device still for a small time interval, the controlled FB peak velocity is approximately increased by a factor of 2 (recall that power is force × velocity).

### 7.3 Power absorption evaluation

To evaluate power absorption, the 9V-DC LEGO wheel is used; hence, no auxiliary sensors are required. Specifically, the 9V-DC motor output voltage (v(t)) is directly measured by the *visualisation Arduino* and employed as a surrogate of power output (p(t)), since $p=v^{2}/R_{t}$, with $R_{t}$ being the load resistance at the output of the 9V-DC motor. Here, it is important to note that, due to uncertainty in the system components and unmodelled losses, it is not possible to obtain $R_{t}$ accurately, and, subsequently, measuring an exact figure of the absorbed power is also not possible. Thus, in this section, a *relative* measure of the average absorbed power that compares the cases with and without latching is used:

(2) $$G_{in}\left[\text{-}\right]=\frac{{\bar{p}}_{c}}{{\bar{p}}_{u}}=\frac{1/t\int_{T_{w}}v_{c}^{2}d\tau }{1/t\int_{T_{w}}v_{u}^{2}d\tau },$$

where ${\bar{p}}_{c,u}$ are the controlled and uncontrolled average absorbed power, and $v_{c,u}$ are the controlled and uncontrolled measured output voltages, integrated over a time window $T_{w}$. Observe that, using the quotient in (2), $R_{t}$ is no longer required.

**Fig. 20:**
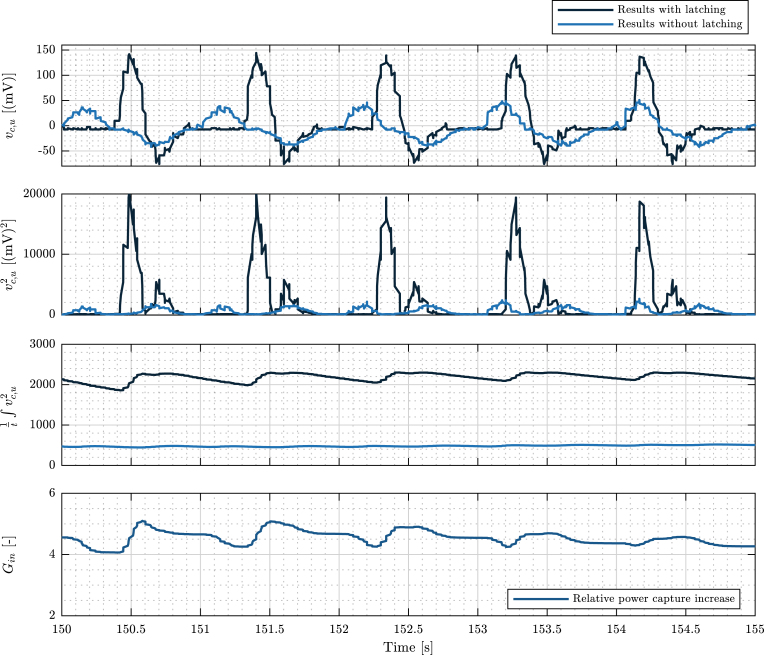
Measured output voltage and relative power absorption evaluation.

The results obtained to validate Tiny-T, in terms of power absorption, are presented in Fig. 20. Observe, first, the measured output voltages $v_{u}$ and $v_{c}$ in Fig. 20.a. Here, it is possible to observe that the peak output voltage is almost three times the peak in the uncontrolled case. Using the measured voltages, it is possible to compute $v_{c,u}^{2}$ (see Figs. 20.b), integrate it and average it (see Fig. 20.c), and use it to evaluate $G_{in}$ (Fig. 20.d). In Fig. 20.d, it is possible to observe that, on average, the power absorption increases by a factor of 4.5 times, illustrating the impact of control on wave energy systems. Remarkably, the increase in power absorption is obtained with a simple half-cycle latching control strategy, which highlights the impact that simple control technology can have on energy absorption for large-scale wave energy systems.

## CRediT authorship contribution statement

**Pedro Fornaro:** Writing – original draft, Visualization, Methodology, Conceptualization. **Jacopo Ramello:** Software, Resources, Methodology, Conceptualization. **Facundo Daniel Mosquera:** Writing – review & editing, Validation, Software, Data curation. **Giuseppe Giorgi:** Supervision. **John Vincent Ringwood:** Writing – review & editing, Supervision, Project administration, Funding acquisition, Conceptualization.

## Declaration of competing interest

The authors declare that they have no known competing financial interests or personal relationships that could have appeared to influence the work reported in this paper.

## Footnotes

1 Here, it is important to bear in mind that results obtained using Tiny-T cannot easily be extrapolated to larger-scale scenarios: While Froude scaling may be preserved, viscous forces are exaggerated at small scales and the Raynolds number (depending on the floating body geometry) changes considerably for different device scales. Also, multiple unmodelled effects appearing due to the tank dimensions (reflexions, shallow water effects, non-vanishing evanescent modes near the wave maker) cannot be predicted..
